# Supplementary material for: Epidemiological profiles and pathogenicity of Vancomycin-resistant Enterococcus faecium clinical isolates in Taiwan
Source: PeerJ. 2023 Feb 23;11:e14859. doi: 10.7717/peerj.14859 (PMC9968458; doi:10.7717/peerj.14859)
Supplement: Supplemental Information 3 [file peerj-11-14859-s003.docx]

Table S3. Primer sequence

| Target gene | Sequence (5’ to 3’) | Product size (bp) | References |
| --- | --- | --- | --- |
| *asa1* | F: GCACGCTATTACGACTATGA  R: TAAGAAAGAACATCACCACGA | 375 | (7) |
| *gelE* | F: TATGACAATGCTTTTTGGGAT  R: AGATGCACCCGAAATAATATA | 213 | (7) |
| *cylA* | F: ACTCGGGGATTGATAGGC  R: GCTGCTAAAGCTGCGCTT | 688 | (7) |
| *esp* | F: AGATTTCATCTTTGATTCTTGG  R: AATTGATTCTTTAGCATCTGG | 510 | (7) |
| *hyl* | F: ACAGAAGAGCTGCAGGAAATG  R: GACTGACGTCCAAGTTTCCAA | 278 | (7) |
